# Characterizing Prostiva^TM^ RF Treatments of the Prostate for BPH with Gadolinium-Enhanced MRI

**DOI:** 10.1100/tsw.2009.4

**Published:** 2009-01-18

**Authors:** Christian Huidobro, Benjamin Larson, Samuel Mynderse, James J. Myers, David Busel, Cristian Acevedo, Thayne R. Larson, Lance A. Mynderse

**Affiliations:** ^1^ Department of Urology, University of Chile, Santiago, Chile; ^2^ Cleveland Clinic Lerner College of Medicine, Cleveland, OH, United States; ^3^ Mayo Clinic Biomedical Imaging Resource, Rochester, MN, United States; ^4^ Urologic Physicians, Minneapolis, MN, United States; Department of Radiology, University of Chile, Santiago, Chile; ^6^ Institute of Medical Research, Scottsdale, AZ, United States; ^7^ Department of Urology, Mayo Clinic, Rochester, MN, United States

**Keywords:** BPH, minimally invasive, TUNA, prostate, MRI, Prostiva

## Abstract

Transurethral needle ablation (TUNA) is an accepted and effective therapy for the treatment of lower urinary tract symptoms (LUTS) due to benign prostatic hyperplasia (BPH). Prostiva^TM^ (Medtronic, Shoreview, MN) is the newest-generation device, which includes a new needle design and radio frequency (RF) generator. This device creates temperatures of 120°C and necrotic lesions in less than 2.5 min. Using previously described techniques, we analyzed dynamic, gadolinium-enhanced MRIs to characterize the ablative properties of the new Prostiva^TM^ RF device.

Ten men with LUTS due to BPH were treated with the standard Prostiva^TM^ manufacturer–recommended protocol. The bladder neck and lateral lobes received treatment based on prostate volume and prostatic urethral length. Gadolinium-enhanced MRI sequences were obtained prior to and 1 week post-treatment. Analyze® software (Mayo Clinic Biomedical Imaging Resource, Rochester, MN) was used to evaluate MRIs. New gadolinium defects were seen in all patients following Prostiva^TM^ treatments. All lesions coalesced within the prostate. No defects were seen beyond the prostate, and the urethra was spared in all patients. The mean volume of necrosis was 7.56 cc, representing a mean of 11.28% of total prostate volume.

Dynamic, gadolinium-enhanced MRIs demonstrate new vascular defects representing necrosis caused by Prostiva^TM^ RF therapy of the prostate. The standard Prostiva^TM^ RF protocol produces lesions that coalesce to create larger lesions in the bladder neck and lateral lobes. Compared to the TUNA® Precision Plus^TM^ device, the ablative lesions appear comparable while produced with a shorter burn time.

## INTRODUCTION

Urology is a field of medicine driven by innovation. The introduction of new techniques, medications, tests, and devices constantly reshapes its daily practice. While federal standards and associations regulate the development, and grant final approval on medical innovations, responsibility falls on the individual physician to understand and implement new practices correctly. In order to do this, urologists need to understand the mechanisms of action not only of drugs, but also of new devices through scientific investigation.

Improved minimally invasive techniques for the treatment of lower urinary tract symptoms (LUTS) due to benign prostatic hyperplasia (BPH) continue to be developed. As these new devices and modifications are introduced, they should be accompanied by sound data to substantiate the scientific merit of the improvement. While clinical results are necessary to prove the effectiveness of these devices, physicians should not treat patients without an understanding of how each device functions. Characterization of the location and extent of necrosis generated by minimally invasive devices for the treatment of LUTS/BPH contributes to their safe and effective implementation into patient care.

Transurethral needle ablation (TUNA) is an accepted treatment of LUTS due to BPH[1,2,3,4,5,6,7,8]. This technology uses radio frequency (RF) to produce therapeutic temperatures and necrosis around the tips of the treatment needles. TUNA allows the surgeon to directly position areas of ablation at desired locations within the prostate[9,10,11,12]. Prostiva™ (Medtronic, Shoreview, MN) is the newest-generation RF device, which includes an improved needle design and RF generator. This device creates temperatures of 120°C and causes necrotic lesions in less than 2.5 min.

The ablative capabilities of the TUNA® Precision Plus™ device have been well documented through the use of dynamic, gadolinium-enhanced MRI[13]. As the Prostiva™ device enters the marketplace, it is vital to ascertain the ablative properties of this next-generation technology within the prostate.

## MATERIALS AND METHODS

Under internal ethics review board approval and with informed consent, a single experienced clinician treated ten men with LUTS due to BPH with Prostiva™ using the manufacturer's recommended protocol. Patients were selected from symptomatic men scheduled for transurethral resection of the prostate (TURP). The treatment regimen was identical to a prior study[13]. Needle insertion depth was determined by prostatic dimensions measured by transrectal ultrasonograpy. The number of treatment planes was calculated based on the prostatic urethral length as measured by cystoscopic evaluation. Periprostatic block anesthesia with 1% lidocane by transrectal ultrasonography was administered to all patients. The bladder neck of each patient was treated with treatment planes centered at the 3, 5, 7, and 9 o'clock positions. Treatment of the lateral lobes was accomplished with needle placements at the 3 and 9 o'clock positions on descending treatment planes of 1 cm towards the verumontanum. Needle burns of 2 min 20 sec with peak temperatures of 120°C were administered according to manufacturer recommendations.

MRI was conducted according to previously described methods[13]. A 1.5-T field strength magnet Siemens scanner (surface coil), gadolinium dynamic enhancement, and 2D spoiled gradient echo sequences (2.5-mm increments) were performed prior to Prostiva™ treatment and 1 week post-treatment for all ten patients. Both axial and coronal images were recorded. Dark areas on these MR images represent nonviable tissue due to decreased uptake of gadolinium. Prior studies have correlated these findings to histological necrosis[14,15].

Analyze® software (Mayo Clinic Biomedical Imaging Resource, Rochester, MN) was used to measure total pre- and post-treatment prostate volumes, and the amount of necrosis as determined by axial and coronal images.

## RESULTS

Ten patients, mean age 62 years (range: 54–69), were treated. The mean pretreatment prostate volume as determined using Analyze® software was 66.33 cc (31.4–127.7 cc) per axial MRI measurement and 67.6 cc (39.5–141.5 cc) per coronal MRI measurement.

The average number of needle sticks was 9.4 (7–12). Each needle stick lasted 2 min 20 sec at a temperature of 120°C. All patients received complete treatment of the bladder neck and lateral lobes (Fig. 1). One patient had a large middle lobe, which demonstrated treatment effect (Fig. 2). No technical difficulties or treatment complications were experienced. The prostatic urethra was preserved in all patients with gadolinium-enhancing tissue present in all images. A Foley catheter was placed if patients could not voluntarily void following the procedure.

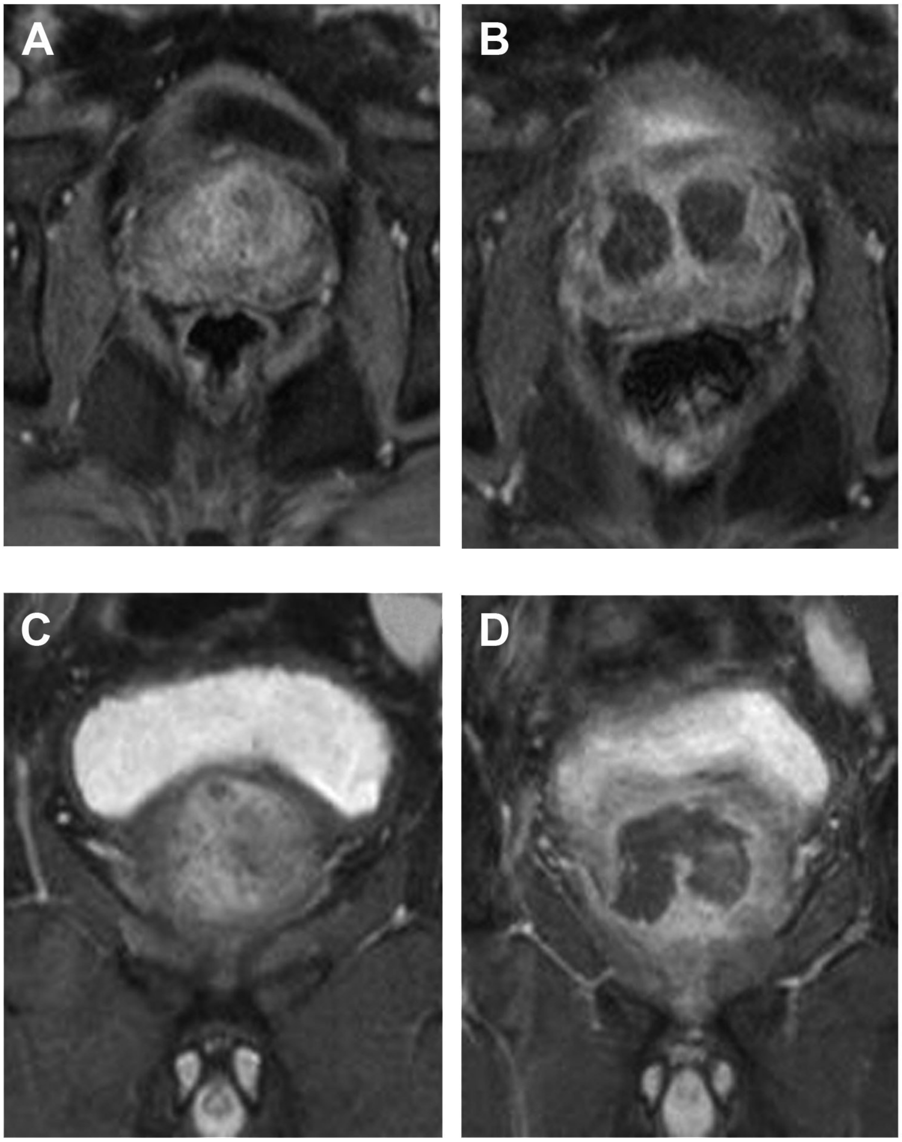

**FIGURE 1.** Pre- (A, C) and post-treatment (B, D) axial (A, B) and coronal (C, D) MRIs of bladder neck and lateral lobes.

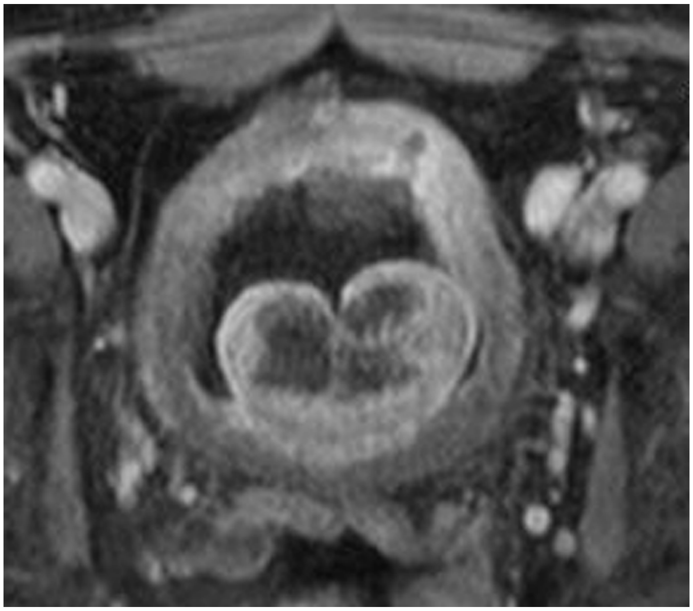

**FIGURE 2.** Axial, post-treatment MRI of large middle lobe, demonstrating gadolinium defect.

Coalesced lesions were observed as a continuous gadolinium defect in all patients (Fig. 3). A representative lesion was rendered in three dimensions using Analyze® software (Fig. 4). Lesion volumes were calculated by integrating individual image necrosis volumes in both axial and coronal planes.

The mean volume of necrosis in the prostate was 7.14 cc (2.84–15.36 cc) by axial assessment and 7.56 cc (3.27–17.32 cc) by coronal assessment. These volumes of necrosis represent a mean of 10.85% (per axial) and 11.28% (per coronal) of total pretreatment prostate volume. The largest lesion ablated was 15.4 cc (per axial) and 17.3 cc (per coronal) in the same patient, representing 12.0 and 12.2% of total pretreatment prostate volume, respectively.

Comparing pre- and post-treatment prostate volumes, as calculated by axial MRI, mean pretreatment prostate volume was 51.04 cc (37.98–65.54 cc) and mean post-treatment prostate volume was 57.58 cc (31.41–82.26 cc). A mean prostate volume increase of 21.47% (3.5–20.3%) 1 week after therapy was observed following treatment with Prostiva™

## DISCUSSION

As new devices emerge in the field of minimally invasive treatment for LUTS due to BPH, treating physicians need to know not only that they are effective, but also how and where they cause treatment effect. In this study, using the Prostiva™ device, gadolinium-enhanced MRIs clearly demonstrated newly generated coalesced gadolinium defect lesions in the prostate of all ten patients. These lesions were not present on pretreatment MRIs and correlated with the location of needle placement in the prostate. Following the manufacturer's recommended protocol (i.e., needle depth, number, and location of "burns"), no areas of necrosis outside of the prostate were noted. There were no gadolinium defects near the rectum or apex, and the urethra was preserved in all cases. As in our prior study, needle ablation was performed from the bladder to the mid portion of the prostate[13]. No burns were done near the apex, according to the protocol. Accordingly, there were no lesions seen near the apex on MRIs. The amount of necrosis and the percentages of total prostate volume ablated will vary depending on the protocol used. This minimally invasive BPH treatment is unique due to the ability of the surgeon to determine the location and extent of treatment using needle ablation.

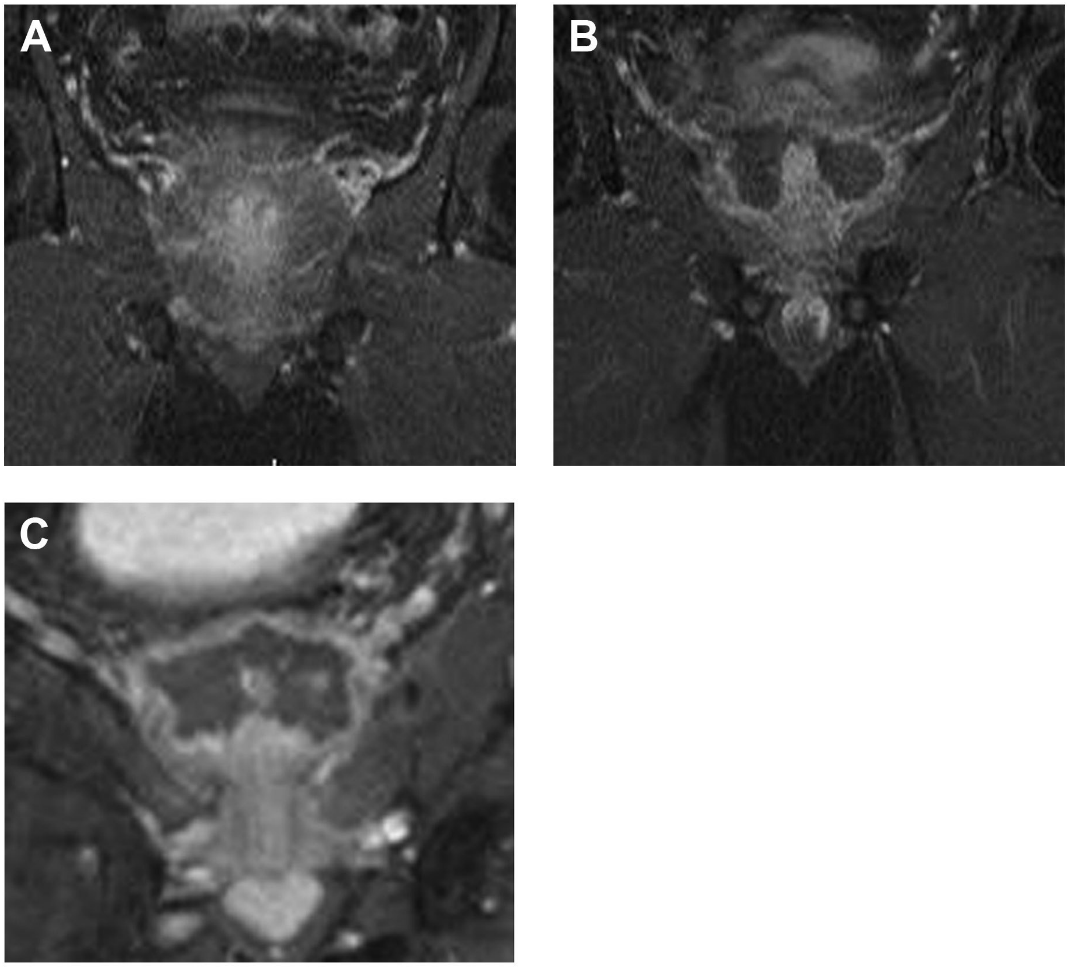

**FIGURE 3.** Pre- (A) and post-treatment (B, C) coronal MRIs of coalesced lesions as a continuous gadolinium defect around bladder neck and mid prostate.

Our prior study of TUNA® Precision Plus™ used the same protocol for 12 patients[13]. While these studies were not designed to compare Prostiva™ to its predecessor, treatments were done by the same physicians using a similar protocol. In the previous study, the necrosis volume measured 8.6% (per axial) and 9.8% (per coronal) of total prostate volume. This is less than the 10.85% (per axial) and 11.28% (per coronal) created by the Prostiva™ device. While no statistical conclusions can be drawn from these data, it appears that the Prostiva™ device is capable of causing at least equal necrosis as the TUNA® Precision Plus™ in less time (2 min 20 sec vs. 3 min). As noted in the previous study, TUNA has the unique advantage over other thermal therapies for LUTS due to BPH in that the amount and location of necrosis can be strategically determined by the physician[13]. The amount of necrosis can conceivably be increased by a larger number of needle sticks into the prostate.

The post-treatment volumes measured in this study demonstrate an increase in prostate volume 1 week after treatment. The significance of this is unknown, but is most likely due to inflammatory response caused by thermal damage of the Prostiva™ therapy. Further studies are needed to quantify this increase in prostate volume after Prostiva™ treatment over time periods longer than 1 week.

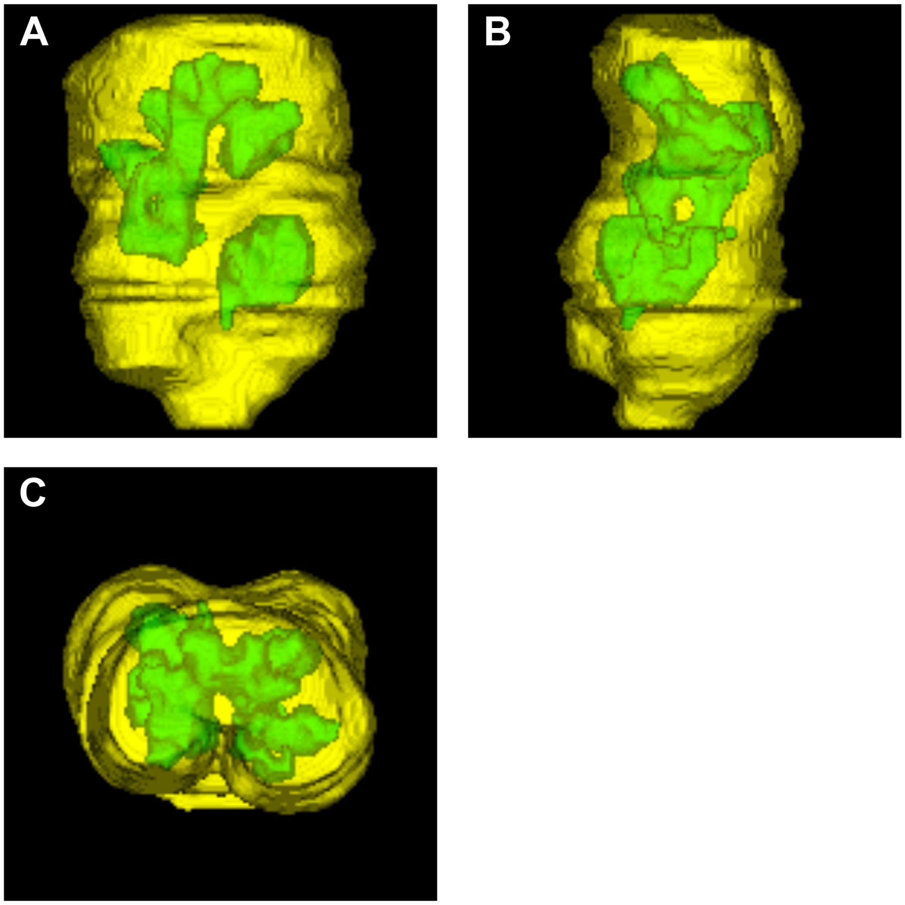

**FIGURE 4.** 3D rendering of the composite lesions using Analyze® software in three projections: anterior/posterior (A), lateral (B), and inferior (C).

## CONCLUSION

This image study characterizes the ablation generated by the Prostiva™ device for the treatment of LUTS caused by BPH. The Prostiva™ device is capable of generating coalescing lesions within the prostate that represent ablation of 11.38% of prostatic tissue. Lesions were located in the bladder neck and lateral lobes in all patients. All gadolinium defects were contained within the prostate using the manufacturer's recommended protocols with strategically placed treatment needles by the surgeon.
